# Upgrading of the L-P Band Cryogenic Receiver of the Sardinia Radio Telescope: A Feasibility Study

**DOI:** 10.3390/s22114261

**Published:** 2022-06-02

**Authors:** Adelaide Ladu, Luca Schirru, Francesco Gaudiomonte, Pasqualino Marongiu, Gianmarco Angius, Federico Perini, Gian Paolo Vargiu

**Affiliations:** 1 National Institute for Astrophysics (INAF), Cagliari Astronomical Observatory, Via della Scienza, 5, 09047 Selargius, Italy; francesco.gaudiomonte@inaf.it (F.G.); pasqualino.marongiu@inaf.it (P.M.); gianmarco.angius@inaf.it (G.A.); gianpaolo.vargiu@inaf.it (G.P.V.); 2 National Institute for Astrophysics (INAF), Istituto di Radioastronomia (IRA), Via Fiorentina, 3513, 40059 Medicina, Italy; federico.perini@inaf.it

**Keywords:** Sardinia Radio Telescope, radio astronomy cryogenic receivers, radio frequency interferences, microwave components for radio astronomy applications

## Abstract

The Sardinia Radio Telescope is a quasi-Gregorian system with a shaped 64 m diameter primary reflector and a 7.9 m diameter secondary reflector. It was designed to operate with high efficiency across the 0.3–116 GHz frequency range. The telescope is equipped with a cryogenic coaxial dual-frequency L-P band receiver, which covers a portion of the P-band (305–410 MHz) and the L-band (1300–1800 MHz). Although this receiver has been used for years in its original design, with satisfactory results, it presents some parts that could be upgraded in order to improve the performances of the system. With the passing of time and with technology advances, the presence of unwanted human-made signals in the area around the telescope, known as radio frequency interferences, has grown exponentially. In addition, the technology of the receiver electronic control system became obsolete and it could be replaced with next-generation electronic boards, which offer better performances both service reliability and low generation of unwanted radio frequency signals. In this paper, a feasibility study for improving the L-P band receiver is discussed, taking into account the mitigation of the main radio frequency interferences. With this study, it is possible to have a sensitive instrument that can be used for scientific research at low frequencies (P- and L-bands), which are usually populated by signals from civil and military mobile communications, TV broadcasting and remote sensing applications.

## 1. Introduction

Radio telescopes are useful sensors that allow us to observe sources in a different way with respect to optical astronomy. Typically, they consist of reflector antennas or a set of multiple connected antennas (i.e., antenna array) which work together as a single system. They collect weak radio waves, amplify them and make them available for the final back-end analysis. These radio waves are emitted by astronomical radio sources in outer space, such as stars, galaxies, black holes, and other objects of the universe. In the world, there are several radio telescopes of different shapes and sizes. Regarding reflector antennas, there is Green Bank Telescope (GBT) with a diameter of 100 m, located in the United States, which was developed to operate in a frequency range between 300 MHz and 116 GHz [1]. China and Australia feature the Tianma telescope (65 m of diameter and 1.25–50 GHz of operative frequency [2]) and the Parkes antenna (64 m of diameter and 0.7–25 GHz of operative frequency [3]), respectively. In Europe, the 100 m Effelsberg antenna (Germany) permits researchers to work at 0.3–96 GHz [4]. 

Italy also has its role in the scientific community thanks to its radio telescopes. The Sardinia Radio Telescope (SRT), a fully steerable multi-reflector antenna designed to operate with high efficiency across the 0.3–116 GHz frequency range [5], represents one of the Italian radio telescopes of ownership of the National Institute for Astrophysics (INAF) and in particular of the Astronomical Observatory of Cagliari (OAC). Its optical design is based on a quasi-Gregorian configuration with a shaped 64 m diameter primary reflector and a 7.9 m diameter secondary reflector, in order to minimize the spillover and the standing wave between secondary mirror and feed [6]. Moreover, the telescope is equipped with a beam wave guide (BWG) room, where there are available four 2.9 m mirrors and one 3.9 m mirror, for a total of four additional focal positions [6]. This optical design allows us to equip up to fifteen receivers on the SRT that can be automatically selected through mechanical systems. One of the most important properties of SRT is the active surface system, which allows us to compensate for the gravitational deformation, the thermal gradients and the pressure of the wind of the 64 m dish (by means of an electro-mechanical control), guaranteeing a high antenna efficiency at high frequencies [7]. Four radio frequency receivers are available at present, covering a portion of P-band (305–410 MHz), L-band (1300–1800 MHz), C-band (5.7–7.7 GHz) and K-band (18–26.5 GHz) [8,9]. Moreover, two new receivers are currently under design and development: a C-band phased array feed (PAF), based on the PHAROS2 project [10,11,12], which covers the whole frequency range between 4 and 8 GHz, and a 7-feeds S-band receiver (3–4.5 GHz) [13,14,15]. Although SRT has been designed for radio astronomy observations up to 116 GHz, currently the maximum frequency of observation is 26.5 GHz. The telescope is involved in research activities such as astronomy, geodesy and space science. SRT also operates in the Very Long Baseline Interferometry (VLBI) network, a technique that performs correlation between data collected by several antennas that observe the same radio source, simultaneously [16,17,18]. Recently, the antenna has also been employed for Space Situational Awareness (SSA) operations [8,19,20,21,22,23,24]. In this kind of applications, the cryogenic coaxial dual-frequency L-P band receiver has been of fundamental importance to cover low frequencies with satisfactory results.

With the aim of maximizing the scientific research results and extending them to new high frequencies (up to 116 GHz) not yet covered by SRT, INAF has recently expressed an interest to upgrade the telescope. For these reasons, a National Operational Program (PON) funding [25] has been allocated to INAF by the Italian Ministry of University and Research, with the aim to install on the telescope four new receivers that operate in Q-band (33–50 GHz) and W-band (70–116 GHz) [25,26,27]. The works relating to the PON project have imposed a stop on the SRT operations and, consequently, the temporary dismantling of the old receivers (P-band, L-band, C-band and K-band [9], respectively). By taking advantage to this, it is possible to study, upgrade, maintaining, testing and refurbishing them in laboratory to verify their actual performances. 

**Figure 1:**
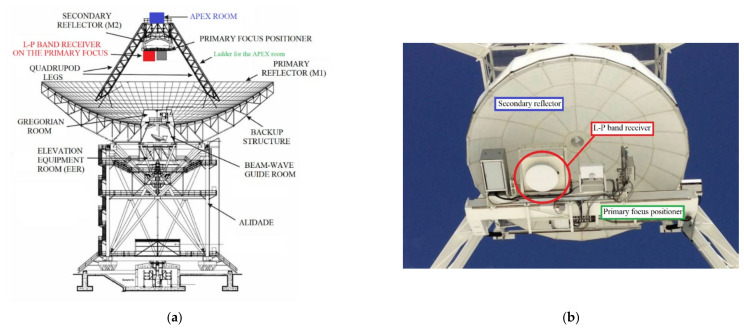
(**a**) Sketch of the Sardinia Radio Telescope (SRT), where the position of the L-P band receiver (primary focus of the telescope), the APEX room and the ladder for the APEX room, are highlighted; (**b**) Photo of the L-P band receiver installed on the primary focus of SRT.

Focusing on the L-P band receiver of the SRT, it is installed on the primary focus of the telescope (see Figure 1a,b) and it permits simultaneous observations in both the P-band and L-band, as well as classical observation at a single frequency [28,29]. Although the receiver has been used in its original design with good results for years, it presents some aspects that could be upgraded in order to improve the performances of the system. With advancing years, the technology used for developing the control system of the receiver became obsolete and it could be substituted with next-generation electronic boards. These new electronic systems offer better performances both service reliability and low generation of unwanted radio frequency signals, better known as radio frequency interferences (RFIs). They represent spurious signals and harmonics from others frequency bands, self-produced signals (generated by the old electronic control system), undesired signals overlapping the receiver band (i.e., the signals of civil and military mobile communications, the TV broadcasting and the remote sensing applications) and out-of-band signals not properly rejected by the microwave filters of the system. Unfortunately, the RFI scenario of the surrounding environment evolves over time and it is worsened with respect to the time when the L-P band receiver was developed and installed on the SRT. For these reasons, a feasibility study is necessary to establish what changes to make to the receiver. These changes regard the replacement of original electronic control system with a next-generation board, which offer better performances both service reliability and low RFIs generation, and the study of the requirements of new microwave filters for high RFIs rejection. These new components will replace the old filters of the receiver, allowing us to cut inoperable frequency sub-bands affected by the RFIs. Furthermore, considering that the RFI scenario could deteriorate further in the next future, it could be necessary to modify the operative frequency of the system with the installation of other filters. Consequently, it is necessary to have the filters block of the receiver in a most comfortable position respect to the primary focus of the telescope, which can be only reached using a lifting platform. For this reason, in the feasibility study presented in this paper, the authors consider to move the filters block in the APEX room of SRT, adequately prepared to accommodate them and accessible by a ladder installed on one of the four quadruped legs (see Figure 1a).

The aim of this paper is the feasibility study for the upgrade, the refurbishment and the improvement of the cryogenic coaxial dual-frequency L-P band receiver. In Section 2, the architecture and design of the original system is described. The study of the receiver criticalities and the workflow for the upgrading feasibility study are addressed in Section 3. The results of this feasibility study are discussed in Section 4 and, finally, the conclusion about this work are presented in the last section.

## 2. Architecture of the Original L-P Band Cryogenic Receiver of SRT

As mentioned in the Introduction section, the L-P receiver consists of in a cryogenic coaxial dual-frequency system, which permits concurrent observations in the P- and L-bands, along with single band observations, in either linear or circular polarization. In particular, it is composed of two coaxial feeds that are designed to work optimally in the range 305–410 MHz of the P-band and 1.3–1.8 GHz of the L-band, respectively [29]. Since SRT is a super-heterodyne system that works at a baseband frequency of 0.1–2.1 GHz [30], a down-conversion system for the radio frequency (RF) signals detected by the L-P receiver is unnecessary.

**Figure 2:**
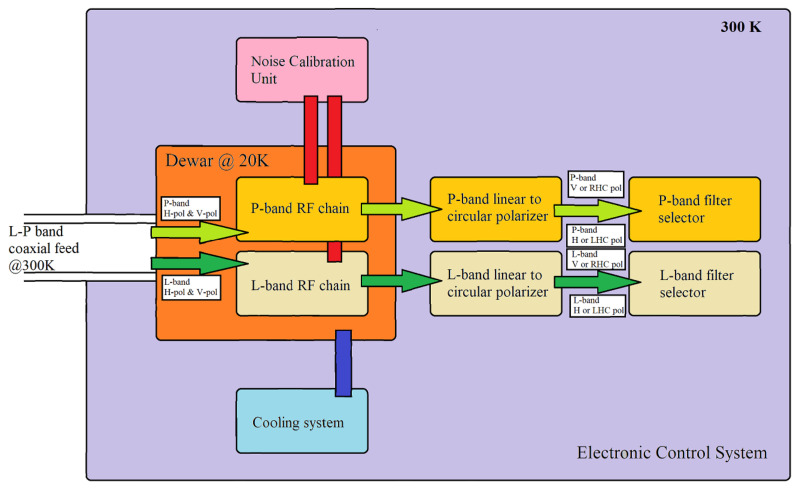
The block diagram of the L-P band receiver of SRT.

**Figure 3:**
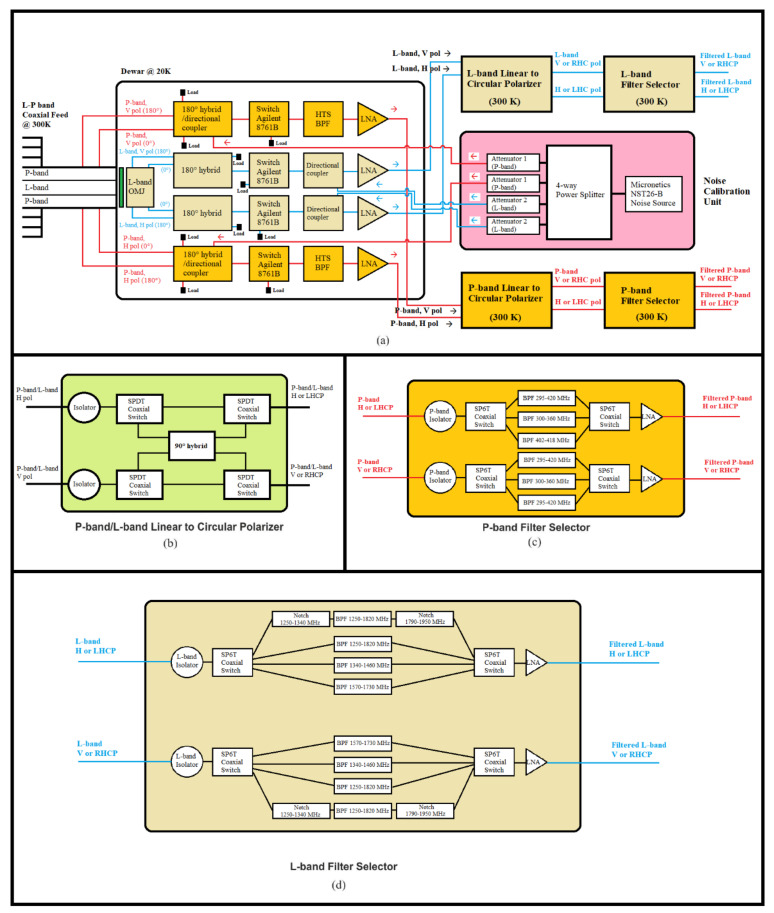
(**a**–**d**) Schematic of the L-P band receiver [29].

A block diagram of the receiver is reported in Figure 2 and a schematic representation of the original receiver front-end, focusing on the microwave components, is shown in Figure 3. All RF and electronic components of the original receiver architecture are integrated into a single box, installed directly on the primary focus positioner of SRT, with a volume of about 3 cubic meters and a weight of about 700 kg [29]. The features of these blocks are summarized below.

Analyzing in detail the block diagram of the receiver architecture (Figure 2), it is composed of:

- The L-P band coaxial feeds, working at ambient temperature (i.e., 300 K);
- The Dewar (or cryostat) block that works at cryogenic temperature of about 20 K;
- The cooling system, which enables the Dewar refrigeration;
- The noise calibration unit, useful for the receiver calibration for both P-band and L-band, thanks to the injection of a noise source (using the broadband microwave coaxial noise source NST26-B model from Micronetics [31]) in the RF signal acquisition chains;
- The P-band and L-band polarizer blocks, which permit us to also obtain the circular polarization;
- The P-band and L-band filter selector blocks, which allow us to select the suitable microwave filter for radio astronomy observations.

**Figure 4:**
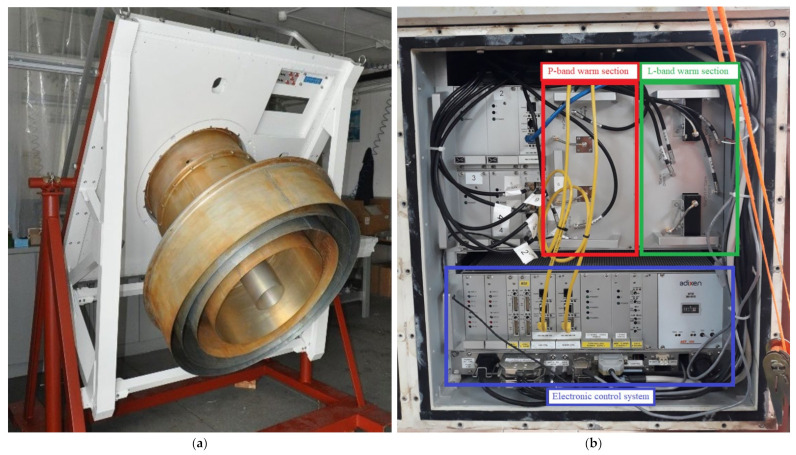
(**a**) Photo of the feed; (**b**) Photo of the warm section (which works at the temperature of 300 K) of the receiver, composed of the linear-to-circular polarizer, the filter selector block and the electronic control system.

All architecture blocks can be managed remotely thanks to a dedicated electronic control system (see Figure 4b), composed of some electronic boards described in detail in [32]. The main electronic board is composed of several digital input/output (I/O) electronic components, in order to create an interface that adds the ability to input and output digital signals in parallel to a computer. The used I/O electronic components are based on the main I/O communication technologies, such as transistor–transistor logic (TTL), inter-integrated circuit (IIC) serial bus, serial communications interface (SCI) bus, controller area network (CAN) bus and serial peripheral interface (SPI) bus. The microcontroller used for the board implementation is the FR50 32-bit RISC Core model from Fujitsu [33]. In particular, the board, interfaced with all receiver components, was programmed with an ad hoc firmware and software in order to manage the cooling system, with the aim to achieve the cryogenic temperature of about 20 K, and supplying the necessary voltage to all active microwave components of the system. The board permits us only to switch on the microwave components, without any control to the voltage levels and the possibility to temporarily switch off the components. However, the board enables us to collect data about the state of the Dewar (the temperature and the pressure) and the state of the active components (i.e., the voltages of the low noise amplifier stages). In this way, it is possible to conduct statistical analysis and have valuable information about the correct functioning of the receiver. Further details about this electronic board are presented in [32].

Focusing on the microwave components of the system, a detailed schematic is reported in Figure 3. The L-P band feeds are realized using a coaxial configuration. In particular, the P-band feed and its ortho-mode junction (OMJ) are designed and realized in a coaxial circular waveguide, with an outer diameter that represents the P-band feed aperture equipped with external corrugations for the radiation pattern improvement, and an inner diameter corresponding to the L-band feed, which is a simple truncated circular waveguide (see Figure 4a). This part of the system works at 300 K. Further details of the feed design are explained in [29].

Continuing with the analysis of Figure 3, the feeds are directly connected to the Dewar (or cryostat) system. This block is directly attached to a cooling system that guarantees to achieve the cryogenic temperature of about 20 K, as shown above in Figure 2. The cryostat size is optimized with the goal to not have too many empty spaces between microwave components of the signal acquisition chain. In this way, the cryogenic temperature can be maintained more easily. On the Dewar block, all RF microwave components that compose the signal acquisition chain for the P-band and L-band channels are installed, respectively (see Figure 3). Regarding the P-band channel, the first component of the RF chain is a cryogenic 180° hybrid with integrated directional coupler [34]. This is a six-port microwave component that is realized using a planar fractal 180° hybrid configuration and a coupled lines directional coupler in cascade on the same layout, in order to significantly reduce its size on the Dewar [34]. Downstream from this component (see Figure 3), there is a commercial coaxial mechanical switch (model Agilent 8761B [35]), provided for the P-band low noise amplifier (LNA) isolation in case of high self-produced RFIs [29]. The next component of the Dewar signal acquisition chain is a cryogenic high-temperature superconducting (HTS) band pass filter (BPF) [36]. This filter is used before the LNA with the aim to reject high RFIs slightly outside the receiver bandwidth, which would be amplified in an undesirable mode by the LNA. The last component (into the Dewar block) of the P-band signal acquisition chain is the cryogenic LNA, with a gain of about 27 dB at the center frequency [37].

As concerns the L-band channel, on the Dewar block there is the ortho-mode transducer as a first component of the RF chain. It consists of a cylindrical ortho-mode junction (OMJ) and two identical 180° hybrid power combiners in a double ridged waveguide [38]. After this component, a commercial switch (model Agilent 8761B [35]) was installed for the same reason described for the P-band channel. The next component is a directional coupler that permits us to inject a noise source in the RF path, useful for the receiver calibration [39]. Finally, the last component of the chain is the L-band LNA, with a gain of about 38 dB at the center frequency [40].

Each output signal from the Dewar enters into the P-band/L-band linear-to-circular polarizer block, respectively (see Figure 3). This block maintains both linear polarizations (i.e., H-pol and V-pol) and, using a commercial 90° hybrid, collects them in right-hand circular polarization (RHCP) and left-hand circular polarization (LHCP).

**Figure 5:**
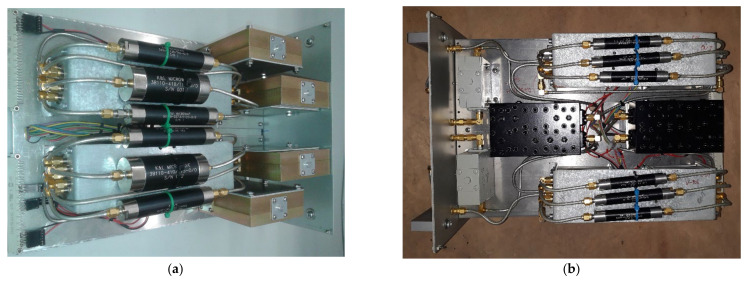
(**a**) P-band filter selector block; (**b**) L-band filter selector block.

The final block of the signal acquisition chain is the filter selector block. Regarding the P-band channel (see Figure 3 and Figure 5a), this section of the receiver front-end includes, besides a microwave isolator as a first component, a coaxial switch (model 87104A from Agilent, Santa Clara, CA, USA [41]) that allows us to select the desired filter and, as last component, a commercial LNA (model ZRL-700 from Mini-circuits, New York, NY, USA) that works at ambient temperature with a gain of about 30 dB [42]. The installed commercial filters are:

- A band pass filter (BPF) centered at 357.5 MHz with a bandwidth of about 120 MHz (model 5B340-357.5/T120-O/O from K&L, Salisbury, MD, USA [43]). It is a five-pole tubular filter with a −3 dB bandwidth from 295 MHz to 420 MHz and an insertion loss less than 1 dB;
- A BPF centered at 330 MHz with a bandwidth of 50 MHz (model 5B340-330/T5O-O/O from K&L, Salisbury, MD, USA [43]). In particular, it is a five-pole tubular filter with a −3 dB frequency response in the frequency range between 300 MHz and 360 MHz, and an insertion loss less than 1 dB;
- A BPF centered at 410 MHz with a bandwidth of 16 MHz (model 3B110-410/T15-O/O from K&L, Salisbury, MD, USA [43]). In detail, it is a three-pole tubular filter with a −3 dB bandwidth from 402 MHz to 418 MHz and an insertion loss of about 1 dB.

All these filters are used for both polarization channels (i.e., P-band H-pol and V-pol), respectively. 

As concerns the L-band filter selector block (see Figure 3 and Figure 5b), it includes a microwave isolator as a first component, a coaxial switch (model 87104A from Agilent, Santa Clara, California [41]) that allows us to select the desired filter and, as last component, a commercial LNA (model ZRL-2150 from Mini-circuits, New York, NY, USA) that works at ambient temperature with a gain of about 25 dB [44]. The available commercial filters, for each polarization channel, are:

- A BPF centered at 1540 MHz with a bandwidth of 520 MHz (model 5B120-1540/T520-O/O from K&L, Salisbury, MD, USA [43]). In particular, it is a five-pole tubular filter with a −3 dB frequency response in the frequency range between 1250 MHz and 1820 MHz, and an insertion loss less than 1.5 dB;
- A BPF centered at 1400 MHz with a bandwidth of 120 MHz (model 5B120-1400/T120-O/O from K&L, Salisbury, MD, USA [43]). In detail, it is a five-pole tubular filter with a −3 dB bandwidth from 1340 MHz to 1460 MHz and an insertion loss less than 1.5 dB;
- A BPF centered at 1655 MHz with a bandwidth of 120 MHz (model 5B120-1655/T120-O/O from K&L, Salisbury, MD, USA [43]). In particular, it is a five-pole tubular filter with a −3 dB bandwidth from 1570 MHz to 1730 MHz and an insertion loss less than 1 dB;
- The combination of three cascaded filters in order to obtain an observation bandwidth in the range between 1300 MHz and 1800 MHz, cutting the main RFIs at 1310–1340 MHz and 1790–1960 MHz. These three filters are: a band rejection (Notch) filter (model 6N45-1320/E62.7-O/O from K&L, Salisbury, MD, USA [43]), a BPF centered at 1540 MHz with a bandwidth of 520 MHz (model 5B120-1540/T520-O/O from K&L, Salisbury, MD, USA [43]) and another Notch filter (model 6NS11-1880/E138-O/O from K&L, Salisbury, MD, USA [43]).

Further details about the architecture of the original L-P band receiver are reported in [29].

## 3. Study of the Receiver Criticalities and Workflow for the Upgrading Feasibility Study

The receiver upgrading feasibility study of this paper considers those technical aspects that can improve the performances of the system. One of them regards certainly the RFIs mitigation, that can derive from the surrounding area, described in Section 3.1, or that can be self-produced by internal components (i.e., the original electronic control system and the feeding process of the active microwave components), aspects addressed in Section 3.2. Finally, some logistical changes are proposed in Section 3.3.

### 3.1. Mitigation of the RFIs Derived from the Surrounding Area

In recent years, as technology advances, the generation of radio frequency and microwave signals has grown exponentially. All this involves a continuous evolution of the RFI scenario, which represents a very relevant issue for radio telescopes. In fact, the periodic monitoring, and the resulting knowledge of unwanted man-made RFI signals, represents a crucial operation for preventing to mask weak cosmic signals detected by the telescopes involved in radio astronomy research activities. Certain radio observatories design and develop ad hoc hardware fully dedicated to RFIs detection [45]; some others prefer to dedicate a low percentage of the observing time with the aim to accomplish the activities of RFIs monitoring. This option has the benefit of taking advantage of the telescope’s high sensitivity to identify the RFIs, but at the cost of taking time to conduct the astronomical research.

**Figure 6:**
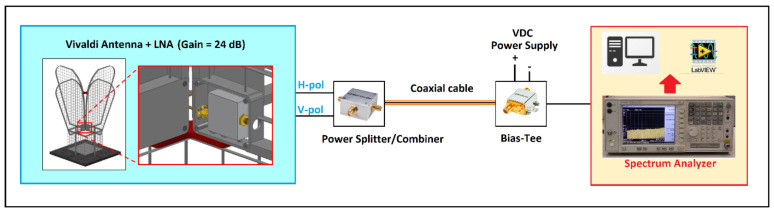
Schematic of the RFI monitoring system based on one of the Vivaldi antennas of the Sardinia Aperture Array Demonstrator (SAD) [45,46].

**Figure 7:**
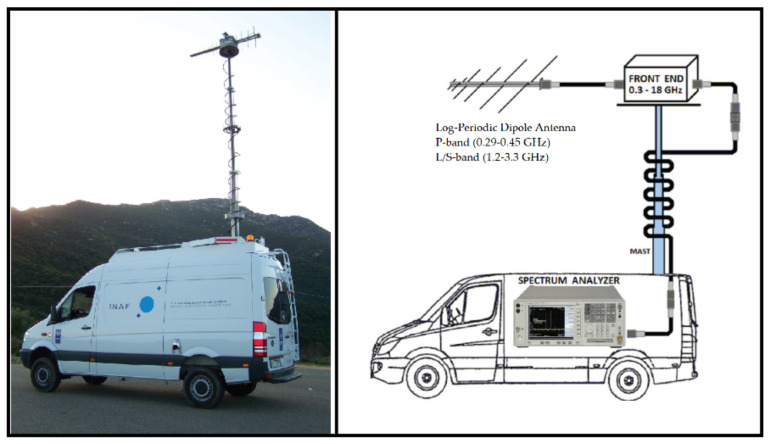
The RFI mobile laboratory [47].

In the case of our radio observatory, other than using SRT for activities of RFIs detection, two ad hoc systems are available:

- One of the Vivaldi antennas of the new Sardinia Aperture Array Demonstrator (SAD) telescope, with its dedicated signal acquisition chain [45,46], is shown in Figure 6. This system is located close to the SRT area and it is designed to cover the frequency range 50–500 MHz, with an antenna gain of about 8 dBi. It represents a useful fixed station, operating automatically and remotely in order to guarantee continuous data acquisition 24 h per day, and 7 days per week. In this way, it is possible to map the RFI scenario around SRT, detecting both continuous and impulsive signals. Further details of the system are described in [45,46].
- The RFI mobile laboratory, depicted in Figure 7, that was designed with the aim to have a very high sensitivity and a large linear dynamic range, without neglecting to limit the generation of self-produced RFIs [47]. The system is equipped with an aluminum retractable telescopic mast that can lift one antenna and can be rotated in azimuth electronically or manually. The whole RF receiving system works up to 18 GHz and its front-end was accurately characterized by using microwave instruments with high performances. Further details are reported in [47]. As concerns the P- and L-bands, the RFI mobile laboratory is equipped with a P-band log-periodic dipole antenna (LPDA) that covers, with a gain of about 12 dBi, the frequency range 0.29–0.45 GHz, and a L/S-band LPDA with a gain of about 11 dBi between 1.2 and 3.3 GHz [47]. This system, being a mobile station, is useful for surveys aimed at verifying the RFIs distribution in the neighborhood of the telescope, with the possibility to choose different locations such as a realistic and more pessimistic view of the expected spectrum receivable by SRT.

When the L-P band receiver was installed on SRT, there was a RFIs situation less critical than now. In fact, the uptake of broadband telecommunications networks (both civilian and military) and third, fourth and five generations cellular phones have led to an increase in the RFIs in the P and L frequency bands. Despite the recurring RFI measurement campaigns performed with the aim to update the RFI maps around the telescope, a new detailed campaign is necessary to determine if there is the necessity to install new microwave filters, for latest RFIs rejection, on the upgraded receiver. These filters are to be engineered to cut all main RFIs with levels of amplitude too high and guarantee a more or less cleaned band of observation.

The new RFI campaign is conducted using the RFI monitoring system based on SAD, for the P-band, and SRT, for both P- and L-bands. Using two different systems, it is possible to compare several data with the goal to identify the vast majority of undesired signals present in receiver bandwidth. In case of detection of new unknown signals, the RFI mobile laboratory can be used in order to investigate the source of them.

**Figure 8:**
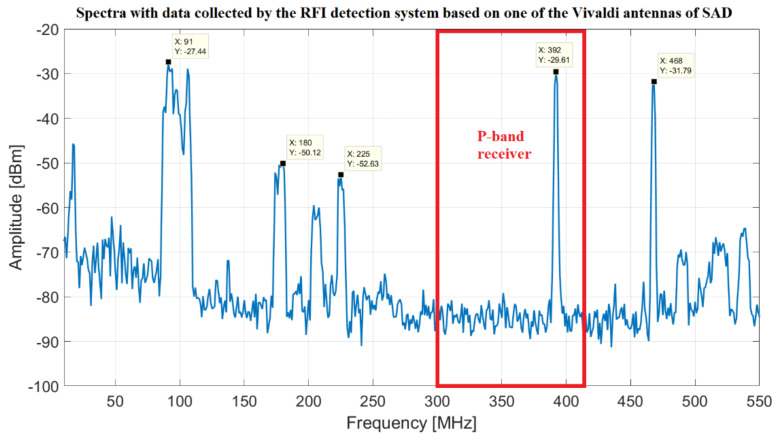
Spectra with data collected by the RFI detection system based on Sardinia Aperture Array Demonstrator (SAD).

As regards the P-band, an explicative spectrum of data collected by the RFI system based on one of the Vivaldi antennas of the SAD telescope [45,46] is reported in Figure 8. In this case, the bandwidth 50–550 MHz is analyzed with the scope to identify the highest level unwanted signals. In this spectrum, some impulsive signals are not visible for reason of graphical representation, but they are equally detected by the system. The measurement campaign performed with this system can be considered a preliminary analysis for studying the situation in the neighborhood of the P-band receiver bandwidth (305–410 MHz squared in red in Figure 8). In the bandwidth 50–550 MHz considered by this system, the significant unwanted man-made signals are imputable to the following:

- FM radio band (88–108 MHz);
- Security services (160–185 MHz);
- Digital Video Broadcasting (DVB) services (203.5 MHz);
- Terrestrial Trunked Radio (TETRA) in use by the Italian Ministry of Defense (385–395 MHz); unfortunately, one of the TETRA stations is installed on Monte Ixi, at about 1 km as the crow flies from SRT;
- Weather balloons (402–405 MHz, but it is also possible to detect them in the radio astronomical service (RAS) band 406.1–410 MHz);
- Sardinia emergency department (460 MHz);
- Radio TV broadcasting signals (470–828 MHz).

According to this preliminary investigation, it is clear that the signal with the highest level of amplitude comes from the TETRA station and it represents a serious limitation for the receiver performances. Furthermore, despite the BPF of the receiver (model 5B340-357.5/T120-O/O from K&L) has a −3 dB bandwidth from 295 MHz to 420 MHz, several signals (i.e., signal at 225 MHz) enter in the signal acquisition chain (smoothed in accordance with the BPF frequency response, but quite high for a highly sensitive system such as SRT) and represent unwanted signals for back-ends. For these reasons, in this feasibility study, we investigate to upgrade the system with a more performant BPF in order to mitigate these painful signals. The features of these new filters, obtained with the upgrading feasibility study, are described in Section 4 (results and discussion).

**Figure 9:**
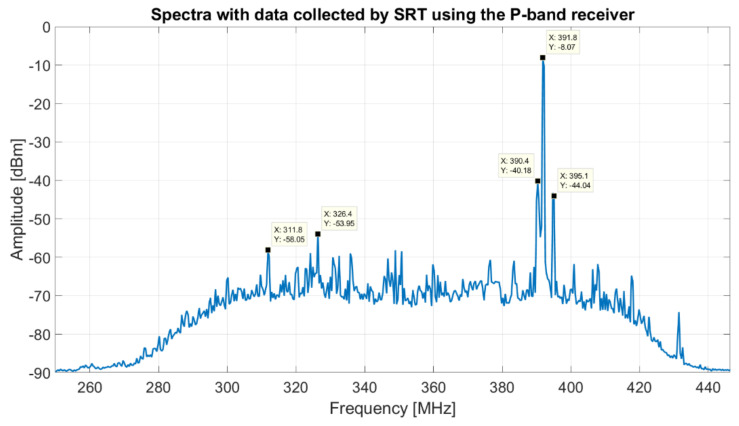
Spectra with data collected by SRT using the P-band receiver, with the selection of the BPF centered at 357.5 MHz with a bandwidth of about 120 MHz (model 5B340-357.5/T120-O/O from K&L), and using the Rodhe and Schwarz FSV40 spectrum analyzer as back-end.

The spectrum of data collected by SRT using the P-band receiver, with the selection of the BPF centered at 357.5 MHz with a bandwidth of about 120 MHz (model 5B340-357.5/T120-O/O from K&L), and using the Rohde and Schwarz FSV40 spectrum analyzer as back-end, is shown in Figure 9.

This spectrum presents a better resolution bandwidth respect to Figure 8 and further RFIs are visible. In particular, in the frequency range between 310 and 330 MHz, there are signals from Italian Air Force. TETRA signals are clearly visible also in this plot, with its high level of amplitude that in certain moments saturates the whole receiver system. Moreover, in the spectrum of Figure 9, are visible several signals with a considerable peak level, such as at 355, 360, 400–401 and 410 MHz, which are self-produced by the electronic control system of the receiver. The mitigation of these RFIs is addressed in the next Section 3.2. 

**Figure 10:**
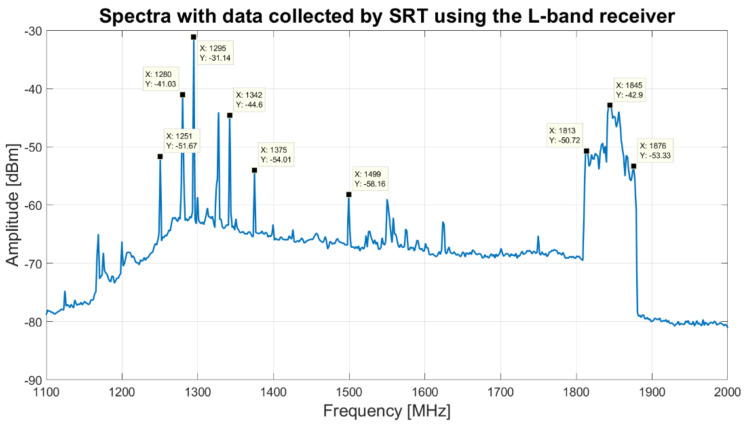
Spectra with data collected by SRT using the L-band receiver, with the selection of the BPF centered at 1540 MHz with a bandwidth of 520 MHz (model 5B120-1540/T520-O/O from K&L), and using the Rodhe and Schwarz FSV40 spectrum analyzer as back-end.

On the other hand, with regard to the L-band, data collected by SRT using the L-band receiver and its BPF 1250–1820 MHz (model 5B120-1540/T520-O/O from K&L), and the Rohde and Schwarz FSV40 spectrum analyzer as back-end, are plotted in Figure 10. The highest RFI signals, into the portion of the L-band covered by the receiver, are due to:

- Italian army radars (1310–1370 MHz);
- Global Positioning System (GPS) bands (the GPS L1 band 1575.42 MHz with a bandwidth of 15.345 MHz, the GPS L2 band 1227.6 MHz with a bandwidth of 11 MHz and the GPS L5 band 1176.45 MHz with a bandwidth of 12.5 MHz);
- Self-produced signal coming from the focus selector of SRT (1499 MHz) [20];
- Radio communication links (1620 MHz);
- Mobile communications (1810–1880 MHz) [48,49].

Analyzing Figure 10, the L-band receiver bandwidth is also busy with disturbing signals. In addition, because the BPF is a five-pole filter and its S21 curve does not present an edge band with very steep drop, several out-of-band signals are detected by SRT and turn out a trouble for the scientific research. Based on these considerations, in this feasibility study we present the features of a possible new filter that can replace the old filter of the receiver filter selector block, in order to improve the whole system. This part is addressed in Section 4 (results and discussion section).

### 3.2. Mitigation of the Self-Produced RFIs

Besides the RFIs from the surrounding environment, unfortunately, there are a number of self-produced RFIs. In Section 3.2.1, the mitigation of RFIs generated by the electronic boards for the remote control of the receiver [32] is addressed. The mitigation of unwanted signals generated by the feeding process of the active microwave components (i.e., microwave switches) is discussed in Section 3.2.2.

#### 3.2.1. Mitigation of the RFIs Generated by the Electronic Control System

The RFIs generated by the original electronic control system are mostly in P-band, such as the signals at 355, 360, 400–401, and 410 MHz, which can be viewed in Figure 9. These electronic boards provide the exact supply voltage for all active components installed on the receiver (i.e., switch, amplifiers, etc.), as well as the monitoring of the system. Unfortunately, in addition to the unwanted signals generation, the original electronic control board does not permit us to temporarily switch off the LNAs, which is an important aspect for isolation in case of high self-produced RFIs [29]. However, as discussed in Section 2, these devices are of paramount importance for the receiver monitoring and they cannot be removed from the system. The issue of RFIs generation can probably be improved with an RF shielding of the original control electronic boards. However, this does not resolve the criticality regarding the LNAs management. For these reasons, in this feasibility study, the replacement of the outdated technology, i.e., the original electronic boards [32], is proposed, considering the latest cutting-edge electronic engineering, with a focus on low RFIs generation components. The new electronic board has to be backward compatible with earlier boards with the aim to maintain the original receiver architecture. 

In the new receivers developed for the PON project [25,26,27], a new electronic board, named Gain Attenuation Intelligent Amplifier (GAIA), is used for the remote control [26]. This board can be considered for the receiver upgrade feasibility study, in order to align the L-P band receiver technology with the PON new receivers. The board is based on a microcontroller (model ATMega2560 from Microchip Atmel [50,51]) and on digital potentiometers (model AD5231 from Analog Devices [52]) designed for biasing, remote monitoring and control of the gate and the drain voltages (V_g_ and V_d_, respectively) of cryogenic LNAs [26]. The GAIA board [26,53] offers ultra-stable biasing of the P- and L-band cryogenic LNAs. GAIA is equipped with a network module based on chip model W5100 from Wiznet [54] for the Ethernet communication. The board allows SPI, IIC and universal asynchronous receiver–transmitter (UART) communication protocols. Further details of the GAIA board are reported in [26,53]. 

**Figure 11:**
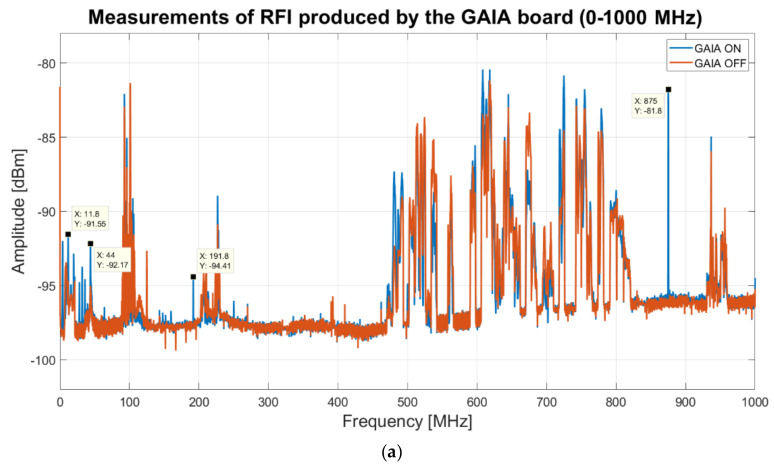

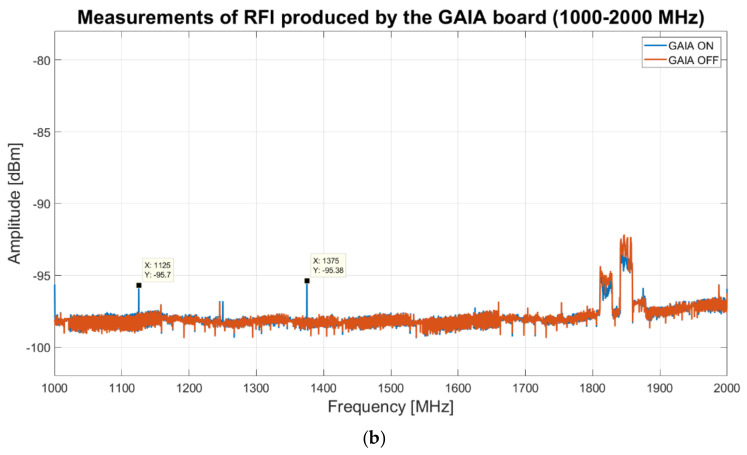
Results of the measurement campaign for surveying the signals generated by the GAIA electronic board [53]: (**a**) frequency range between 0 and 1 GHz [53]; (**b**) frequency range between 1 and 2 GHz [53].

A dedicated RFI measurement campaign, with the goal to study the signal emission of the GAIA board, is presented in [53]. The measurements are performed in an outdoor environment, in open field. The measurement system is composed of an electric field probe connected to a spectrum analyzer (model FSV40 from Rohde and Schwarz). For detecting impulsive signals, the instrument is setup in clear/write mode with resolution bandwidth of 1 MHz and sweep time of 100 microseconds [53]. This analysis did not reveal the presence of impulsive signals generated by the board. Furthermore, the spectrum analyzer is setup in average mode with span equal to 1 GHz, resolution bandwidth of 200 kHz and 5000 resolution points, for detecting continuous signals [53]. The results of the measurements from 0 to 1000 MHz (including the interesting P-band for our application) are shown in Figure 11a and the results for the bandwidth 1000–2000 MHz (L-band) are plotted in Figure 11b [53]. 

Analyzing the plot of Figure 11a, it is visible that the GAIA board generates narrowband signals at 4, 12, 32, 36, 192, and 875 MHz with regard to the P-band [53]. This last RFI represents the highest of signals gave off by GAIA in P-band, but it does not represent an issue for the receiver, since it is far from the P-band receiver bandwidth (i.e., the bandwidth of the P-band feed is 305–410 MHz). Concerning the L-band, the GAIA board emits signals at 1125 and 1375 MHz (see Figure 11b) with low level of amplitudes [53]. These signals do not represent a problem for the receiver, because they are located in the edge of the receiver bandwidth and, in the case of the signal at 1375 MHz, in a zone populated by other RFIs from Italian army radars. In Figure 11a,b, it is possible to see several signals from the surrounding area, such as FM radio signals (88–108 MHz), Radio TV broadcasting signals (470–828 MHz), mobile communications signals (940–960 MHz and 1810–1880 MHz [48,49]), which have nothing to do with the GAIA board. In fact, these signals occur both when the board is powered on (blue curve) and when the board is powered off (red curve). On the basis of these results, it can be stated that the GAIA board is suitable for the remote control system of a sensitive radio astronomy receiver, such as our L-P band cryogenic receiver.

#### 3.2.2. Mitigation of RFIs Generated by the Feeding Process of the Active Microwave Components

**Figure 12:**
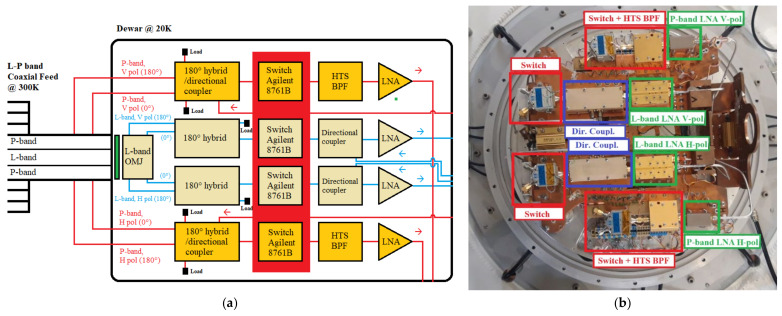
(**a**) Block diagram of the Dewar L-P receiver; (**b**) Photo of the Dewar block.

Regarding the unwanted signals generated by the feeding process of the active microwave components of the receiver, presuming that they are chosen from the state of the art with a low unwanted signals generation, most of them cannot be eliminated. In any case, considering the use and performances of the receiver in its period of service and the future GAIA electronic board installation, some active components can be removed. One type of them is the commercial cryogenic coaxial mechanical switch (model Agilent 8761B [35]), highlighted in Figure 12a and in the photo of the Dewar block (Figure 12b). The receiver was equipped with these switches in order to isolate the LNAs in case of high unwanted or self-produced high signals (i.e., SRT should be equipped in the future with a transmitter for deep space and near-earth applications [29]). The role of these switches of Figure 12 can be executed directly by the new GAIA control board. It can remotely power off the LNA alimentations, guaranteeing a high protection from high power levels of self-generated RFIs, making useless the presence of these switches.

**Figure 13:**
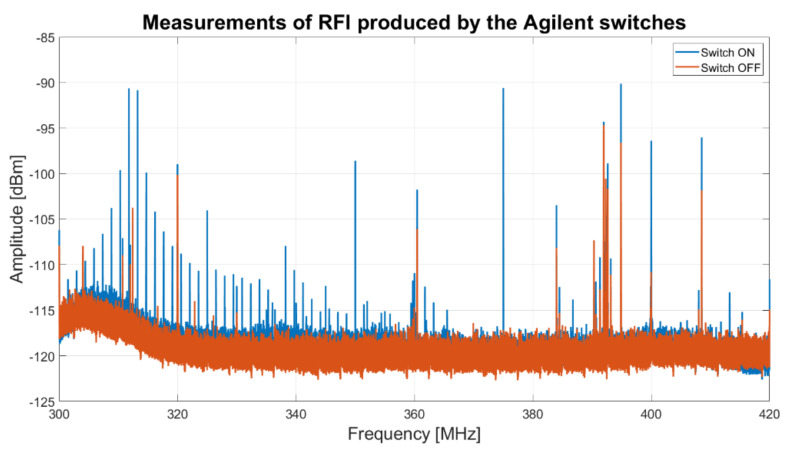
Results of the measurement campaign for surveying the signals generated by the Agilent 8761B switches.

An RFIs measurement campaign is performed in order to establish the amount of signals generated by these components. In particular, the receiver feed is covered with a metal cap and an electric field probe connected to a spectrum analyzer (model FSV40 from Rohde and Schwarz) is used for performing the measurement. The instrument is setup in average mode, with a span of 130 MHz (start frequency at 300 MHz and stop frequency at 430 MHz) and a resolution bandwidth of 50 Hz. The results of these measurements are reported in Figure 13. The plot shows a high number of unwanted signals in the frequency range 300–350 MHz, when the switches are powered on (blue curve), with respect to the situation when the switches are powered off (red curve). Consequently, in order to reduce the self-produced RFIs, these switches can be eliminated from the Dewar block and the GAIA board can be exploited to execute the functions of LNA protection, which were provided by these switches in the original design of the receiver. 

### 3.3. Logistical Changes

The L-P receiver was installed on the primary focus positioner of SRT, as mentioned in the Introduction and shown in Figure 1. This optical position of the telescope represents a critical aspect when talking about receiver maintenance operations. In fact, it is possible to reach the primary focus of the telescope only using lifting platform, with all the attendant limitations (i.e., discomfort for disassembly of receiver components, use only in good weather conditions, etc.). For this reason, in this feasibility study, we consider maintaining, on the receiver block installed on the primary focus of SRT, only the essential components, such as the feed, the Dewar block with its cooling system, the linear-to-circular polarizer, the noise calibration unit and the electronic control system (see these blocks in Figure 3). The filter selector block will be transferred in a dedicated rack installed on the APEX room (see Figure 1). The APEX room is accessible thanks to a ladder installed on one of the four quadruped legs. In this way, in case of receiver malfunction, it is possible to act with maintenance operation analyzing all components of the filter selector block that work at ambient temperature, and only secondarily moving on to analyze the Dewar block with its components. Furthermore, it is possible to install new microwave filters easily, which could be necessary to reject new possible RFIs or to experience new temporary observation modes. In this manner, basically, it becomes a more resilient and practical system.

## 4. Results and Discussion

On the basis of results concerning the RFI analysis of Section 3, it appears that there are several unwanted signals that can be rejected with targeted changes in the receiver. In this upgrade feasibility study, some hardware changes for RFIs mitigation are proposed, such as the replacement of original electronic control system with a next-generation board and the removal of unused microwave components (i.e., the switches of the Dewar block). In addition, the microwave filters of the receiver need to be replaced with more performant filters. The requirement for the design of these components is the attenuation of at least 30 dB for the highest RFIs present in the receiver bandwidth (i.e., TETRA signals, mobile communications signals and Italian army radars signals).

### 4.1. New Filters Requirements for the P-Band Channel

**Figure 14:**
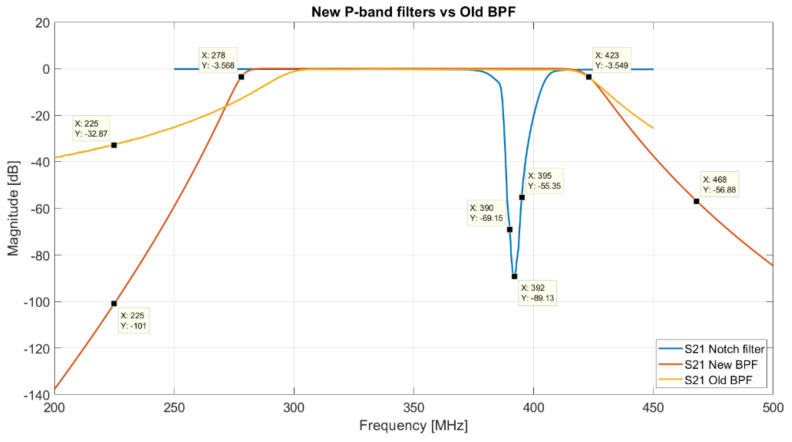
Comparison between the S21 parameter of the old P-band BPF (orange curve), centered at 357.5 MHz with a bandwidth of about 120 MHz (model 5B340-357.5/T120-O/O from K&L) and the new ideal filters (Notch filter and new BPF). The Notch filter (blue curve) rejects the bandwidth 385–395 MHz (TETRA signals of Figure 9) and the new BPF (red curve) has a −3 dB frequency response between 280 and 420 MHz.

Starting from the analysis of the P-band (see Figure 8 and Figure 9) and considering, in particular, the larger bandwidth that the receiver can offer, the most important need is rejecting the TETRA signals (385–395 MHz), which turn out to be the highest unwanted signals in this receiver bandwidth. This depends on the fact that one of the TETRA stations is installed on Monte Ixi, at about 1 km as the crow flies from SRT. The possible solution to this problem is the design and development of an ad hoc band-stop filter (Notch filter). In this way, it is possible to attenuate the frequencies within 385–395 MHz range (stopband) while passing all other frequencies unaltered. A feasibility study to determine the best features of the Notch filter is conducted using the Matlab software and the ideal frequency response of this kind of filter is shown in Figure 14 (blue curve). In detail, this filter attenuates of about −70 dB at 390 MHz, −89 dB at 392 MHz and −55 dB at 395 MHz. At these frequencies, the TETRA signals measured by SRT, using the old BPF (model 5B340-357.5/T120-O/O from K&L), have levels of amplitude of about −40, −8 and −44 dBm, respectively. Because of the installation of the new Notch filter, these TETRA signals are almost completely cut down, thus allowing a cleaner band of observation.

The Notch filter can be cascaded with the BPF. Considering that the old BPF (model 5B340-357.5/T120-O/O from K&L) is a five-pole filter, it has a S21 curve (orange curve of Figure 14) with an edge band with very soft drop, several out-of-band signals (such as the signals at 225 and 468 MHz of Figure 8) are detected by SRT and turn out a trouble for the scientific research. In fact, for radio astronomy applications, it is necessary to consider at least the −30 dB filter frequency response, in contrast to engineering projects that take into account the −3 dB response. This is due to the fact that the highly sensitive radio astronomy tools integrate received signals in the observation bandwidth and they can detect RFIs (although they are outside the −3 dB bandwidth, with an engineering perspective, of the microwave filter) can assume comparable amplitude with real signals. For this purpose, a new BPF can be designed and developed with an ideal frequency response (i.e., S21 parameter) similar to the features calculated with the Matlab software and shown in Figure 14 (red curve). In detail, the new BPF can have a −3 dB bandwidth slightly larger than the old BPF bandwidth (280–420 MHz vs. 300–420 MHz), but with an edge band with a very step drop (such as −101 dBm vs. −32 dBm at 225 MHz).

On the basis of the receiver usage experienced during the years, consideration of the P-band high-temperature superconducting (HTS) BPF [36] (see Figure 12) can be given. Typically, a filter is not used before the LNA because its insertion loss worsens the temperature of the whole system. However, since the P-band is populated by several RFIs also slightly outside the receiver bandwidth, the HTS BPF was used to reject them. Further details of this filter are proposed in [29,36]. Even though it features high performances in terms of band rejection, the HTS filter has the disadvantage of working only at cryogenic temperature. Consequently, when the receiver is at a temperature of 300 K, the filter behaves like a 50 Ohm load. This aspect represents a limit for the P-band receiver, which can be used only at system temperature of about 20 K, excluding the possibility to perform application at ambient temperature (i.e., radar observation at 300 K, etc.). If the scientific community has the need to use the L-P receiver also at ambient temperature, it is possible to replace the HTS BPF with a new cryogenic BPF in accordance with the features calculated with the Matlab software and shown in Figure 14 (red curve).

### 4.2. New Filters Requirements for the L-Band Channel

**Figure 15:**
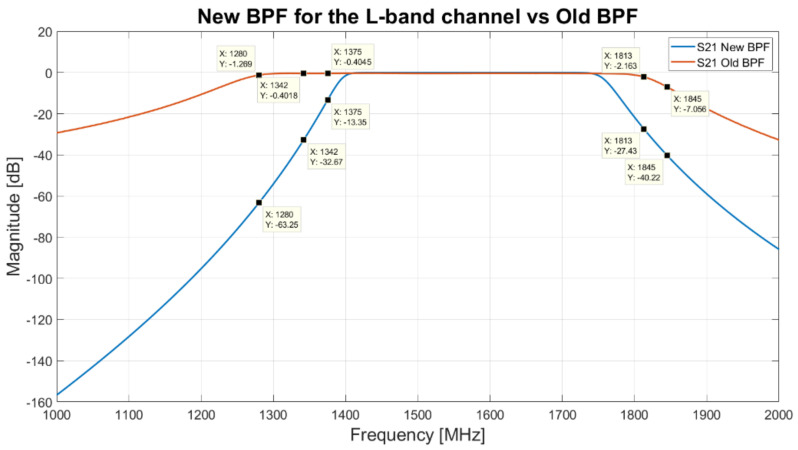
Comparison between the S21 parameter of the old L-band BPF (red curve), centered at 1540 MHz with a bandwidth of 520 MHz (model 5B120-1540/T520-O/O from K&L) and the new ideal BPF (blue curve). It has a −3 dB bandwidth between 1400 and 1750 MHz.

Regarding the broadest portion of the L-band that the receiver can cover (the original −3 dB bandwidth was 1300–1800 MHz), from the analysis of Figure 10, it emerges that this band can be reduced in order to cut troublesome RFIs that reside on sideband. The comparison between the S21 parameter of the new BPF, obtained with Matlab simulations, and the old BPF (model 5B120-1540/T520-O/O from K&L), installed on the receiver, is shown in Figure 15. Thanks to the features of the new BPF, it is possible to attenuate adequately the mobile communications signals (1810–1880 MHz [48,49]) and the Italian army radars signals (1310–1370 MHz). In particular, the new filter attenuates of about −13 dB at 1375 MHz, −32 dB at 1342 MHz and −63 dB at 1280 MHz (Italian army radars signals that can be displayed in Figure 10), while the old BPF attenuates of about −0.4 dB, −0.4 dB and −1.3 dB, respectively. Better results are also obtained for mobile communications signals (shown in Figure 10), such as at 1813 MHz and 1845 MHz, where the new filter attenuates the signals of about −27 dB and −40 dB, compared to −2 dB and −7 dB with the old filter attenuation, respectively.

The analysis of the P-band and L-band channels is conducted considering the broadest bandwidth possible for the receiver, according with the features of the front-end described in Section 2. On the other hand, thanks to the movement of the receiver filter selector block in the APEX room, it is possible to install, at any time, new filters on the receiver that cover a portion of these broadest receiver bandwidths to suite the requirements of the scientific researchers.

## 5. Conclusions

A feasibility study for upgrading the original design of the cryogenic coaxial dual-frequency L-P band receiver of SRT is presented. Although the receiver was used for years in its originally design, with good results, it presented some aspects that could be upgraded in order to improve the performance of the system. In recent years, as technology advances, the generation of radio frequency and microwave signals has grown exponentially. This aspect involves a continuous evolution of the RFI scenario, which represents a very relevant issue for radio telescopes. In particular, the P-band is increasingly populated with signals from the Italian Ministry of Defense (385–395 MHz), the Sardinia emergency department (460 MHz) and the Radio TV broadcasting (470–828 MHz), as well as the signals from mobile communications (1810–1880 MHz), Italian army radars (1310–1370 MHz), GPS systems (L1 band 1575.42 MHz with a bandwidth of 15.345 MHz, L2 band 1227.6 MHz with a bandwidth of 11 MHz and L5 band 1176.45 MHz with a bandwidth of 12.5 MHz) and radio communication links (1620 MHz), which have invaded the L-band. In addition, several self-produced RFIs are generated (both in P- and L-band) by the electronic boards for the remote control of the receiver.

In order to improve the system performance, in this paper, we presented a feasibility study for some hardware modifications of the receiver front-end. In particular, the new BPF features for the P-band and the L-band channels is proposed. These features are obtained using the Matlab software and they are compared with the features of the previous BPFs installed on the receiver. The results shown that, with new filters with these characteristics, it is possible to reject the unwanted signals with the highest levels of amplitude. In addition, the original electronic control system is replaced with an electronic board named GAIA, which also permits the remote control of the LNAs receiver. This board is characterized from the point of view of RFIs emission, which turns out to be much lower than that of old electronic boards used in the original design of the receiver. 

## Data Availability

Not applicable.
